# Study on the heterogeneity of China’s agricultural economic growth in the context of temperature shocks

**DOI:** 10.1038/s41598-022-11877-3

**Published:** 2022-06-22

**Authors:** Bin Yuan, Yuhu Cui, Xueye Wang, Hanxiao Xu

**Affiliations:** College of Management, Ocean University of China, Qingdao, 266100 China

**Keywords:** Climate-change adaptation, Climate-change impacts, Climate-change policy, Environmental economics

## Abstract

Under the background of the new development concept, compared with the absolute impacts, the relative impacts of climate change on agricultural growth deserve more attention. Based on the data from China for years 1991 and 2018, this paper uses historical fluctuations in temperature within cities to identify the heterogeneous effects on aggregate agricultural outcomes during farming and fallow periods. The results show that: first, as temperature rises reduce the economic growth rate of each agricultural sector, and the areas that are relatively vulnerable (i.e., areas where disposable income of farm households is below the sample mean) are more significantly affected by the negative impact of temperature rise; second, the impact of temperature rise on agricultural economic growth is mainly concentrated in the farming period, while the marginal damage of temperature rise is on a decreasing trend; third, the heterogeneous impact of temperature rise on agricultural economic growth during the agricultural fallow period is also not negligible. At the same time, its impact on agricultural economy is still in the primary stage, that is, its marginal damage tends to increase with the increase in temperature fluctuation. These results inform identifying the climate’s role in agricultural development and provide a theoretical and operational perspective for further optimizing the adaptive policy systems. With wide coverage of adaptive technology, we should pay more attention to the even distribution of technological dividends and continuously improve the coping ability of vulnerable groups.

## Introduction

As a backbone industry that alleviates poverty in vast rural areas and creates wealth for farmers, agriculture could act as a ‘‘safety net” in times of disaster that provide security and income to the populations, especially poor^1^.However, as one of the most vulnerable sectors to higher temperatures, more than 50 million hectares of farmland suffered from meteorological disasters a year in China, and the fluctuation of grain production rose by about 18% according to incomplete statistics^2,3^. With profound implications posed by climate change, all countries, organizations, and individuals are actively exploring effective coping strategies to mitigate the adverse effects of climate change. The Chinese government has adopted a "two-wheel drive" program including mitigation and adaptation: In terms of mitigation, a string of policies has been made to curb and reduce greenhouse gas emissions (e.g., transformation and upgrading of traditional industries, renewable energy-based clean development mechanism)^4,5^. In terms of adaptation, a series of operational policies such as promoting the construction of infrastructure and ecological engineering has been provided for relevant producers to reduce vulnerability, alleviating adverse effects of climate change^6^. Nonetheless, because the costs of environmental change are unlikely to be evenly distributed across individuals within a given population, a policy may generate an uneven distribution of benefits across individuals if the climate change delivers uneven quantities of marginal damage and or the benefits from an incremental improvement in the vulnerability differ across individuals^7^. Therefore, in the context that China is about to enter the “post-poverty era”, more clearly understanding the impact of climate change on agriculture and its heterogeneity could not only provide theoretical reference for the promotion of high-quality agricultural development, but also help to design better policies.

In recent years, with the increasing attention to climate change, economists and ecologists have focused on quantifying various climatic effects on economic activities, especially agricultural production^8,9^. In terms of agricultural yields and productivity^10^, adaptive behavior of farmers^11^, technological innovation^12^and other outcomes, a wide array of detailed and in-depth analyses has been performed. Notably, despite of the fact that existing studies have investigated temperature changes from various forms (e.g., accumulated temperature) based on the absolute change of temperature^13^, both the regionalism of climatic conditions and the seasonality of agricultural production were ignored. Specifically, earlier studies in this area typically capture temperature effects during the growing season; On the other hand, due to the differences in marginal impact of climate change stemming from the various vulnerabilities of entities, the costs and benefits generated are unevenly distributed across individuals within a given population^14,15^. Therefore, the research on the heterogeneity of the economic consequences caused by climate change is always controversial, underscoring the need for more rigorous strategies and detailed analyses to estimate. All the same, some scholars have argued that the poor populations would be harmed more from incremental changes in climate conditions^16^. On the one hand, with a higher share of the agricultural economy, the underdeveloped regions have greater vulnerability to climate change compared with developed regions. Therefore, global warming is more likely to cause more damage to poor countries^17^; on the other hand, differences may also arise because the underlying stock of climate and/or defensive investments differ across populations (e.g., due to differences in the technological and capital endowments). Faced with long-term temperature shifts, various and effective adaptive behaviors would be adopted to address the negative effects of climate change in developed regions^18,19^. In summary, the existing studies have focused more on the impact of climate change or temperature shocks on a specific aspect or field of study, lacking some comprehensiveness and spatial and temporal heterogeneity. Therefore, this study is an innovative study based on the above-mentioned aspects: firstly, it is based on comprehensiveness, and considers the real development situation in China and changes the research object from the previous "stock" of agricultural economic output to "incremental” and considers the impact of temperature shocks on China's overall agricultural economy and the growth of various sectors in a comprehensive perspective. At the spatial level, we take the historical average temperature of different regions in China as the benchmark and re-evaluate the temperature change in each region; at the same time, at the temporal level, we consider the different seasonal differences in China and examine the impact of temperature shocks on the agricultural economy from two dimensions: the farming period and the fallow period. In cases where it is believed that costs of climate change are distributed unevenly, it is important to identify which of these underlying sources of differences in benefits is driving the uneven effect so that the possible impacts on growing economic gap across regions can be avoided and/or addressed through policy^20,21^. We also contribute to the literature by exploring effects on income and inequality in host regions, which can provide policy references on the possibility of addressing relative poverty.

Against this background, by capturing the heterogeneous and long-run effect across cities, we consider the current state of climate in China as an example and analyze its impacts on growth of agricultural economy^22^, while providing a precise reference for the formulation of policies combining the climate change fight and relative poverty reduction. Compared with existing papers, the contributions to this strand of the literature can be summarized as follows: First, we aim at quantifying the effects of higher temperatures on numerous dimensions of agricultural economies. While farming output contractions appear to be part of the story, we also find potential effects of higher temperatures on forestry, animal husbandry, and fishery, respectively. Second, in terms of differences in changing range and individual adaptability, we provide a general framework for examining the sources of heterogeneity in marginal impacts of climate change. Third, we also contribute to the literature exploring adverse effects of climate change in growing and slack seasons. What’s more, we have examined whether higher temperatures appear to have persistent or temporary effects on agricultural economy by looking at multiple lags of temperature, based on a specific spatial pattern at the city level and a long study period of 20 years. These results inform policy designs that can aid farmers’ adjustment process toward better adapting to climate change. We believe this paper is useful because it makes some headway in exploring potential effects of shifts in climate and provides the underlying sources—for both policymakers and economists—of heterogeneity in economic impacts of temperature shocks.

The remainder of the paper is organized as follows. The second section presents a theoretical framework that was composed of the heterogeneous and long-run effects of higher temperatures on agricultural economy. The third section introduces the data and provides a description of the data for analysis, while fourth section describes the estimation strategy, presents the estimated impacts of longer run temperature shifts, and considers several robustness checks. Results of the regression analysis and discussions are presented in fifth section, while conclusions and policy implications are inferred in last section.

## Theoretical framework and methodology

### Theoretical analysis

**Figure 1:**
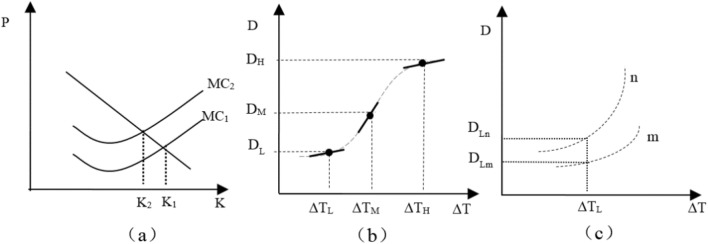
Differences in the marginal damage of temperature changes under different scenarios.

Based on natural and social sciences, there is a growing consensus of the negative effects of climate change on agricultural production. Taking temperature changes as an example, rising temperatures will affect the photosynthesis and disease resistance of crops, thereby reducing agricultural yield^23^. In this situation, producers who are of a rational turn of mind, will readjust the allocation of production factors in term of the current environmental and technical conditions, to get the most out of money. As shown in the left panel of Fig. 1, *ΔT* presents the range of temperature change and *K* presents the factors input under the condition of maximizing. With the changing range of temperature rising from *ΔT*_*L*_ to *ΔT*_*M*_, the negative impact on agricultural production is further strengthened, and the curve of marginal cost on factors also moves from *MC*_*1*_ to *MC*_*2*_. Meanwhile, in cases where it is believed that farmers inevitably reduce the input of elements, the level of agricultural output will also decline and deviate from the optimal input of elements.

It is worth noting that heterogeneity in temperature effects may stem from differences in the changing range and individual adaptability, holding other factors constant. Specifically, if effects are nonlinear with respect to higher temperatures, then two individuals facing various changing range of temperature will experience different marginal damages, even if they are identical in terms of all other factors that determine vulnerability; In cases where the deviation of the temperature from the historical average reaches *ΔT*_*M*_, the marginal temperature effects on agricultural output reaches the maximum and then gradually weakens, shown in the middle panel of Fig. 1; Alternatively—or in addition, even if two individuals are identical in terms of higher temperatures, heterogeneity also may stem from differences in individual adaptability that controls how higher temperatures translates into damages, such as defensive investments (e.g., building a canal.) or avoidance behavior (e.g., adjusting planting structure)^24,25^. For example, to alleviate the shocks of climate change, farmers could not only adopt a drought-tolerant crop varieties and high-efficiency micro-irrigation technologies, but also introduces agricultural crisis management^26^. What’s more, the unfavorable impacts of the past can also be addressed by changing farming methods^27^. In term of husbandry and fishery, a set of technology portfolios including heat-resistant livestock breeds, improvement of the feed structure and sources and wastewater purification, could be used to purify the water environment^28^. As illustrated in the right panel of Fig. 1, under the same temperature change (in *ΔT*_*L*_), the differences in individual adaptability led to relatively larger temperature effects for household (in *n*), coinciding with the opening gap in agricultural yield levels between households (in *n*) and households (in *m*) households.

### Empirical framework

Following the derivation in Bond^29^, this paper estimates a more general econometric model for identifying effects of temperature in the context of a dynamic panel growth regression. To begin, construct a following production empirical framework, according to the existing paths and mechanisms of the impact of temperature changes on agricultural production:

1 $$Y_{it} = e^{{\beta \vartriangle T_{it} }} A_{it} K_{it}$$

2 $$\Delta A_{it} = g_{i} + \gamma_{0} \Delta T_{it} + \cdots + \gamma_{n} \Delta T_{it - n}$$

where Y_it_ represents the aggregate agricultural output measured by the crop’s yields; K_it_ represents the production factors; A_it_ represents the factor productivity. We assume that A_it_ evolves according to a generalized version of the dynamic process specified in Eq. (2) with *n* lags, i.e., which means that the growth rate of *A* affected by the current and lagged temperature; *∆T* measures the changes in temperature, *β* is the effect of temperature on the average agricultural output; *γ* measures the effects on agricultural productivity. *g*_*i*_ is the average growth of agricultural output when little changes in climate is apparent. After taking logs in Eq. (1) and including the cumulative effect of temperature rise and capital accumulation on output, the following dynamic growth model can be obtained:

3 $$y_{it} = \beta_{0} \Delta T_{it} + A_{it} + k_{it} + \alpha_{1} y_{it - 1} + \cdots + \alpha_{n} y_{it - n} + \beta_{1} \Delta T_{it - 1} + \cdots + \beta_{n} \Delta T_{it - n} + \varepsilon_{it}$$

By adding *n* lags of past output, the formula (3) allows output to depend on n lags of temperature and adding an error term. In cases where the external market environment remains unchanged, substituting formula (2) into the first differenced version of formula (3), we further have a dynamic panel estimation equation of the form:

4 $$\begin{gathered} \Delta y_{it} = g_{i} + \alpha_{1} \Delta y_{it - 1} + \cdots + \alpha_{n} \Delta y_{it - n} + (\gamma_{0} + \beta_{0} )\Delta T_{it} + (\gamma_{1} + \beta_{1} - \beta_{0} )\Delta T_{it - 1} \hfill \\ \;\;\;\;\;\;\;\;\; + \cdots + (\gamma_{n} + \beta_{n} - \beta_{n - 1} )\Delta T_{it - n} - \beta_{n} \Delta T_{it - n - 1} + \Delta \varepsilon_{it} \hfill \\ \end{gathered}$$

To capture the effect of temperature change on agricultural output, we assumed that output growth is in steady-state, and thus rewrite the *∆y*_*it-n*_ and *∆T*_*it-n*_ terms as *∆y*_*it*_ and *∆T* terms, respectively; *ρ* is defined as the estimated coefficient of the effect of temperature change on agricultural output. With relabeling coefficients on *T*, the impact of temperature changes on agricultural output can be obtained by solving the formula (4):

5 $$\Delta y_{it} = {{g_{i} } \mathord{\left/ {\vphantom {{g_{i} } {(1 - \alpha_{1} - ... - \alpha_{n} }}} \right. \kern-\nulldelimiterspace} {(1 - \alpha_{1} - ... - \alpha_{n} }}) + \left[ {{{\sum\limits_{j = 0}^{p + 1} {\rho_{j} } } \mathord{\left/ {\vphantom {{\sum\limits_{j = 0}^{p + 1} {\rho_{j} } } {(1 - \alpha_{1} - ... - \alpha_{n} )}}} \right. \kern-\nulldelimiterspace} {(1 - \alpha_{1} - ... - \alpha_{n} )}}} \right]\Delta T_{i}$$

To simplify the problem, considering the influence of the current temperature change yields (The effect of temperature change is simply $\left[ {{{\sum\limits_{j = 0}^{p + 1} {\rho_{j} } } \mathord{\left/ {\vphantom {{\sum\limits_{j = 0}^{p + 1} {\rho_{j} } } {(1 - \alpha_{1} - ... - \alpha_{n} )}}} \right. \kern-\nulldelimiterspace} {(1 - \alpha_{1} - ... - \alpha_{n} )}}} \right]$, which is identical to $\left[ {{{\sum\limits_{j = 0}^{p + 1} {\gamma_{j} } } \mathord{\left/ {\vphantom {{\sum\limits_{j = 0}^{p + 1} {\gamma_{j} } } {(1 - \alpha_{1} - ... - \alpha_{n} )}}} \right. \kern-\nulldelimiterspace} {(1 - \alpha_{1} - ... - \alpha_{n} )}}} \right]$, since the β terms all cancel.):

6 $$\Delta A_{it} = g_{i} + \gamma_{0} \Delta T_{it} ,\quad y_{it} = \beta_{0} \Delta T_{it} + A_{it} + k_{it} + \varepsilon_{it}$$

To find the effects, abbreviating the formula (6) shows that:

7 $$\Delta y_{it} = g_{i} + (\gamma_{0} + \beta_{0} )\Delta T_{it} - \beta_{0} \Delta T_{it - 1}$$

From the above formula, the effects of temperature change are identified through the examination of effects on production and productivity growth. However, as note by Bond, the separate effects may be heterogeneous. In other words, in cases where the temperature returns to a normal level, the average effect on output will be reversed. For example, although the rising temperature will reduce agricultural yields, agricultural output recovers to normal levels when the temperature returns to pre-rising value. In contrast, the temperature shocks on growth rate of agricultural productivity have always been apparent for long-term, even if the temperature returns to its prior state. The above reasoning means that the changes in temperature may lead to permanent backwardness of the agricultural economy in regions.

### Empirical model

Based on above analysis, this paper constructs the following benchmark panel regressions to identify the impact of rising temperature on agricultural economic growth:

8 $$g_{it} = \theta_{i} + \theta_{rt} + \sum\limits_{j = 0}^{L} {\rho_{j} \Delta T_{it - j} + \varepsilon_{it} }$$

In formula (8), *θ*_*rt*_ represents the time fixed effect, including dummy variables and differences between regions; *θ*_*i*_ represents the regional fixed effect; *ε*_*it*_ is the error term; *∆T* represents the degree of temperature change (tem.) including up to *L* lags of temperature to better capture the dynamics of these temperature effects. Based on the previous theoretical analysis, the prerequisite conditions that all coefficients of yield lags are equal to zero, must be met for the econometric analysis by using formula (7). Therefore, based on examining the correlations between agricultural economic growths in different time periods, this article further takes the linear relationship between lags of growth and random disturbance terms into consideration. For robustness test, we use instrumenting for *∆y*_*it—1*_ with further lags of output growth.

In addition, it is note that the costs of environmental changes depending on the vulnerability of crops and degree of environmental changes, are unlikely to be evenly distributed across populations. On the one hand, the effects are nonlinear with respect to changes in temperature. The rising temperature has coincided with the increasing marginal damages; on the other hand, the vulnerability is positively correlated with sensitivity towards environment and adaptability measures adopted by humans. Compared with the former, the latter is more referential for the formulation of relevant policies by no means. Research has revealed that the climate change has been a long-lasting trend. With a high income and many assets, farmers can effectively alleviate the negative impact of external factors on production through pre-event and post-event defensive investment. Comparatively, impoverished farmers with a lower income are more vulnerable towards external impact^30,31^.

On that basis, to better identify the heterogeneity and sources of effects on agricultural output caused by temperature changes, this paper introduces the temperature changes interacted with relative vulnerability and change into the benchmark panel regressions^32^. As a result, a heterogeneous estimation model of the impact of temperature changes on agricultural economic growth can be obtained: In Eq. (9), this paper adopts per capita disposable income as a measure of vulnerability. The relatively vulnerable area is defined by dummy variables. In other words, the sample area where the per capita disposable income is lower than the average is defined as relative fragility (for simplicity, expressed here by “*poor*”), and thus assume that poor = 1. On the contrary, it is 0. Similarly, concerning the degree of temperature changes, this paper defines the sample area where the current degree of changes is larger than the average of samples as extreme climate changes (expressed here by “*worse*”), and assumes that worse = 1. On the contrary, it is 0.

9 $$g_{it} = \theta_{i} + \theta_{rt} + \sum\limits_{j = 0}^{L} {\rho_{j} \Delta T_{it - j} } + \sum\limits_{j = 0}^{L} {\rho_{j} \Delta T_{it - j} \cdot poor + \sum\limits_{j = 0}^{L} {\rho_{j} \Delta T_{it - j} \cdot worse + \varepsilon_{it} } + \varepsilon_{it} }$$

## Data source and model calibration

### Definition and correction of key variables

#### Definition of explained variables

With the main aim to identify the heterogeneous effects of temperature fluctuations on agricultural growth, this paper chooses the net growth of gross output of farming, forestry, animal husbandry, sideline production and fishery in the sample areas as the explanatory variables instead of taking the output or yield of a single agricultural product as the dependent variable. The gross output of farming, forestry, animal husbandry and fishery are respectively expressed in logarithmic form, while the price index of agricultural production materials in each region is deflated to eliminate the effect of price changes on the output growth. At the same time, based on the differences in the sensitivity and vulnerability of different types of crops and animals to temperature rise, this paper further examines the impact of temperature fluctuations on the growth of four agricultural sectors, namely farming, forestry, animal husbandry and fishery (referring to the main statistical methods and calibers advanced by statistical departments, the gross output value output value involved in this article only includes four categories: Farming, forestry, animal husbandry, and fishery, excluding auxiliary activities. Specifically, farming refers to the cultivation of various crops, which including the cultivation of grains, beans, potatoes, cotton, oil crops, sugar, tobacco leaves, vegetables, horticultural crops, fruits, and nuts, etc.; Forestry includes the cultivation of trees (excluding tea, mulberry and orchards), the harvesting and transportation of wood and bamboo, and the collection of forest products; Animal husbandry includes livestock raising and grazing, poultry raising, hunting, and raising of wild animals; Fishery includes the breeding, fishing of aquatic animals and seaweed plants, to identify the differences comprehensively and accurately in the impact of temperature fluctuations on agricultural growth.

#### Correction of explanatory variables

The previous research at home and abroad has revealed clearly that most scholars have not fully considered the regionality of climate and the seasonality of agricultural production in their studies on the impact of temperature fluctuations on agricultural production and have only focused on the absolute value of the annual average temperature in each region as the independent variable. However, due to the differences in geographic location and climatic conditions, change in temperature varies from region to region. For example, high temperatures above 38 degrees centigrade (abbreviated here as °C) in summer that are commonly seen in southern China, may cause great losses in agricultural production in northern areas. More importantly, instead of showing the same upward trend, temperature fluctuations caused by climate change are highly complex and diverse, and it is very common to see high temperatures in summer and frost in winter in the same region. Therefore, measuring temperature fluctuations based on annual average may overlook the differences between seasons and blur their effects on agricultural production, leading to biased estimation result**s**^33^**.** Based on this, this paper takes the historical average temperature of the sample area as the benchmark and recalibrates the regional temperature fluctuations.

In this paper, *T*_*i*_ is a benchmark for the temperature, expressed by the average regional temperature during 1951–1980, the temperature change(*∆T*_*ij*_) is defined as the gap between current (*T*_*ij*_) and benchmark (*T*_*i*_)temperature in period j of region *i*, which is shown as: *∆T*_*ij*_ = *T*_*ij*_*-T*_*i*_. The criterias are that: on the one hand, with a random time period that used as the benchmark group for regression analysis, we can use the benchmark time period to calculate average regional temperature under the condition that the estimation coefficients of the remaining time periods are all significantly negative; on the other hand, according to a string of literatures on temperature change, we have found that the time periods of the benchmark temperature are various, and most scholars argued that optimal intervals were 15–30 years^34^.

Therefore, considering the data availability, this article uses the average temperature during 1951–1980 as a benchmark for the temperature. In addition, to capture the impacts of temperature fluctuations on agricultural production during different time periods in a more precise manner, a year is divided into two periods, namely the farming period and the fallow period, which are based on the cropping conditions of each region in China. The farming period for single cropping areas is from May to October and the fallow period from November to April, while the farming period in double cropping areas is from April to November and the fallow period from December to March.

### Data source

The data used in this paper come from the “China City Statistical Yearbook”, which mainly covers data of 131 prefecture-level cities in 31 provinces, autonomous regions, and municipalities directly under the central government (With limited data availability, a total of 256 valid sample cities were obtained after removal of the invalid samples. Due to space limitations, the article does not list all sample cities.), except for Hong Kong, Macao, and Taiwan over a period from 1991 to 2018. The number of sample prefecture-level cities cover in the yearbook accounts for 47.1% of the total number of prefecture-level cities in China in 2018, and their gross agricultural output accounts for about 43.7% of the national gross agricultural output in that year, indicating that the prefecture-level cities are representative as a sample. The data on the gross agricultural output of farming, forestry, animal husbandry and fishery are mainly obtained from the “China Rural Statistical Yearbook” as well as the statistical yearbooks of each prefecture-level city and of their provinces. The variables such as monthly temperature fluctuations and per capita disposable income of rural households in each region are mainly obtained from the “China City Statistical Yearbook”, the statistical yearbooks of each prefecture-level city and its province, as well as relevant statistical bulletins and related public government information. This paper replaces the missing data with the mean values of the pre and post periods of the sample data, and the descriptive statistics of the specific variables are shown in Table 1.

**Table 1:** Variable descriptive statistics.

Variable name	Obs	Mean	Std. Err	Min	Max
Gross agricultural output value (million yuan)	3799	12.846	1.638	7.66	17.205
Output value of farming (million yuan) Forestry output value	3799	12.142	1.687	4.984	16.627
Output value of forestry (million yuan)	3799	9.081	1.970	0.418	13.796
Output value of animal husbandry (million yuan)	3799	11.620	16.20	6.086	16.070
Output value of fishery (million yuan)	3799	9.491	2.854	0.305	16.122
Annual average temperature fluctuations (°C)	3799	0.772	1.444	− 5.400	5.400
Average temperature fluctuations during the farming period(°C) PEperiod	3799	0.414	4.102	− 5.733	5.733
Average temperature fluctuations during the fallow period (°C)	3799	1.177	1.837	− 6.450	7.383
Degree of relative vulnerability	3799	0.503	0.501	0	1
Degree of relative change	3799	0.509	0.499	0	1

**Figure 2:**
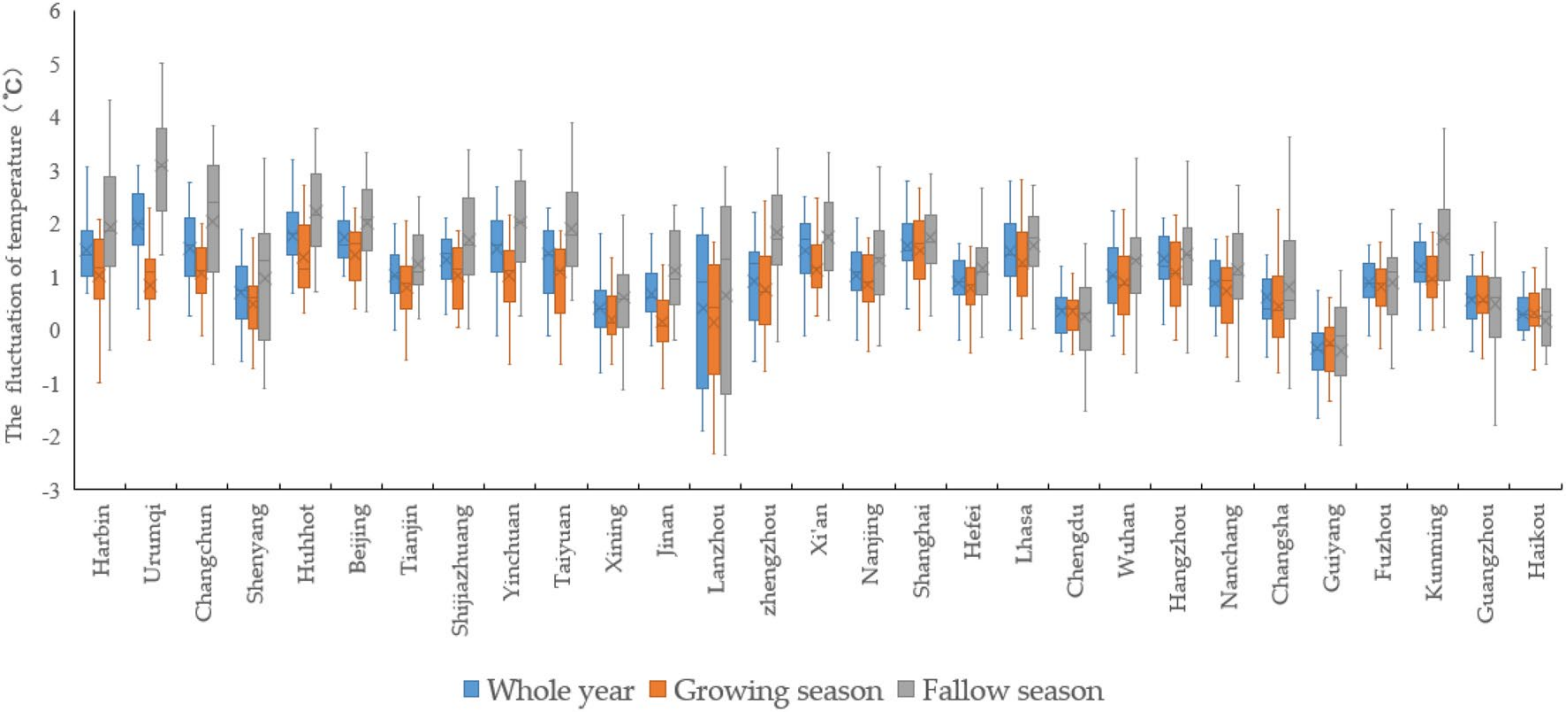
The temperature rises in China's major provincial capitals from 1991 to 2018.

**Figure 3:**
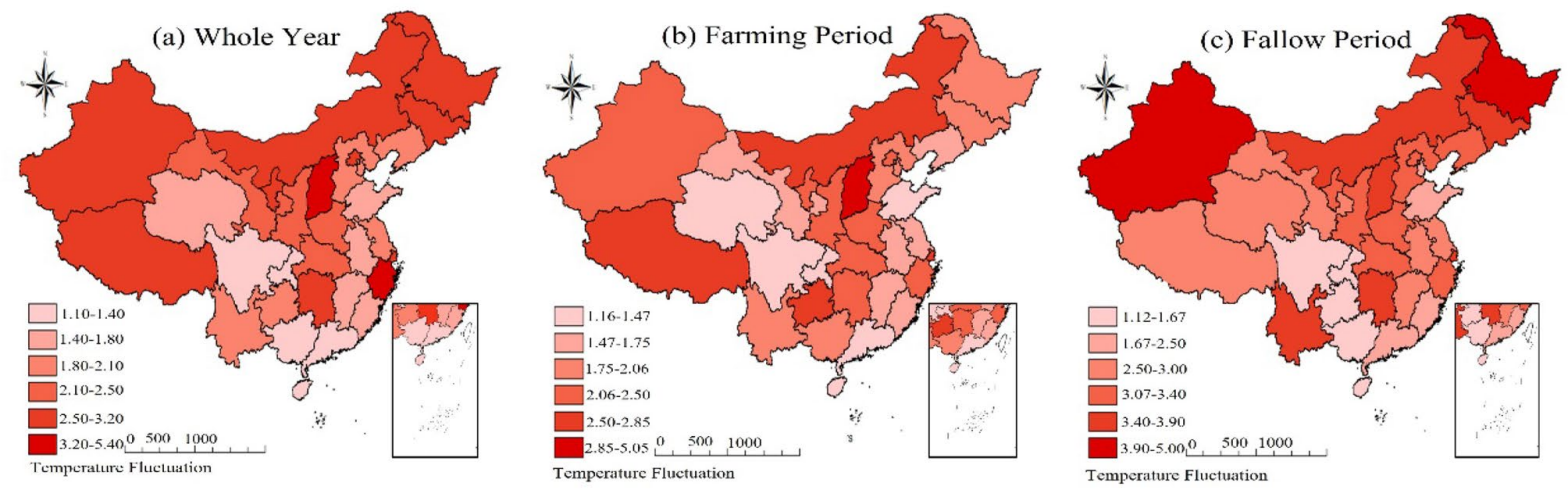
Distribution of high temperature fluctuations in main provincial cities in China (regional maximum temperature—regional historical average temperature).

**Figure 4:**
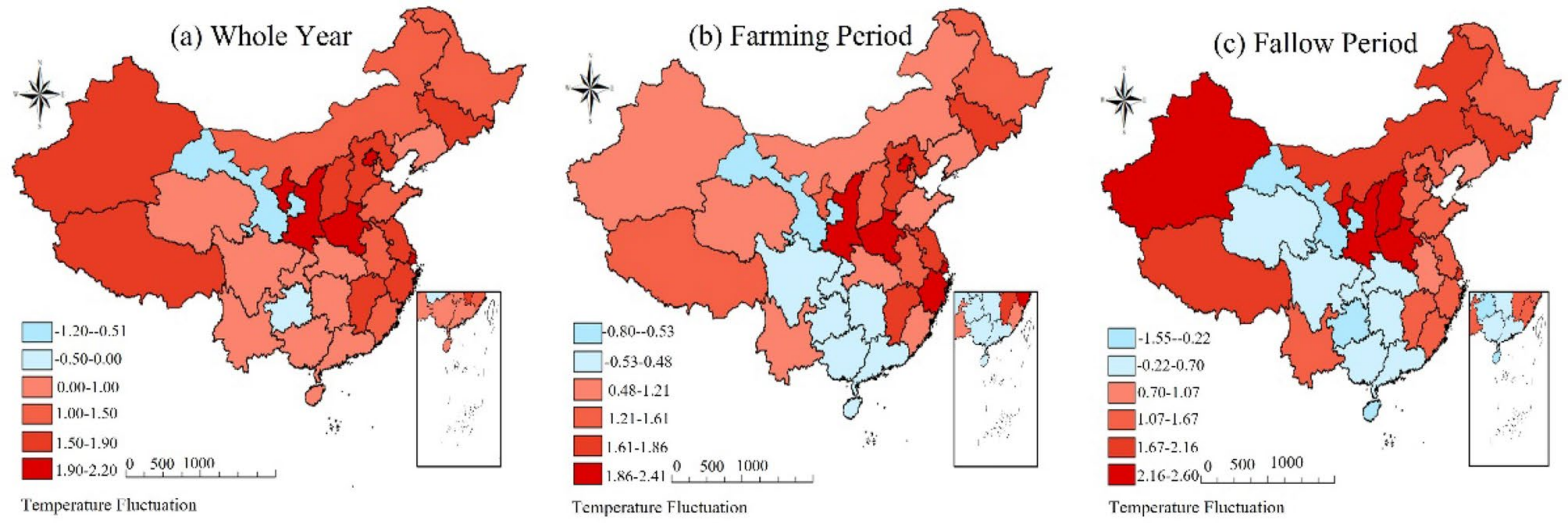
Distribution of low temperature fluctuations in main provincial cities in China (regional minimum temperature—regional historical average temperature).

As shown in Table 1, at the level of China's agricultural production value, both China's total agricultural production value and the production value of each sector have been on an upward trend in the last 30 years. It is worth noting that China's agricultural structure is still dominated by traditional field crops, as evidenced by the higher average value of output corresponding to the agricultural sectors of plantation (12.142 million yuan) and pastoralism (11.620 million yuan), and the lower average value of output corresponding to forestry (9.081 million yuan) and fisheries (9.491 million yuan). In terms of temperature fluctuations in China, the annual average temperature in China increased by about 0.77 °C compared to the same period in history, and the temperature changes were mainly concentrated during the farming period, i.e., from winter to spring each year. Specifically, the average temperature rises of 0.41 °C during the farming period is significantly lower than the annual average. In contrast, the average temperature change during the farming idle period was more pronounced, with an average temperature increase of 1.177 °C. Further combined with the statistical map of temperature in major provincial capitals (Fig. 2), although most regions show a clear upward trend in temperature throughout the year, there are still some periods, especially during the agricultural leisure period, when the average temperature shows a significant decrease. At the same time, since China's climate type distribution is more diverse, according to Fig. 3, for high temperature fluctuations, most of northern China fluctuates more significantly, while Fig. 4 is dominated by low temperature fluctuations, with weaker fluctuations in middle and southern China. In addition, as the latitude of the region decreases with the average temperature, the magnitude of temperature change increases significantly (Figs. 3 and 4 are supported by ArcMap 10.8.1 software, map templates refer to the following link-China Meteorological Administration Public Weather Service Center: https://weather.cma.cn/web/channel-32.html). In the case of cold-temperate regions such as Harbin and Urumqi in the north, the magnitude of temperature change in different years and periods is significantly higher than that in tropical regions such as Haikou and Guangzhou in the south. At the same time, the range of annual average temperature variation in each region is significantly narrower compared to the variation of temperature during the farming and leisure periods. In summary, it is necessary to examine the effects of temperature rise on agricultural production from two time periods, the farming period, and the idle period, respectively.

### Calibration and correction of the estimation model

Based on the above-mentioned theoretical model Eq. (4), before the estimation of the econometric model, the correlation between the agricultural growth with lags needs to be tested to determine whether the growth with lags shall be included in the regression model. The results of the correlation coefficients are shown in Table 2, where a significant correlation is found between the lags of growth in different agricultural sectors. To be more specific, there is a significant correlation between current growth and growth with 4 lags. There is a significant correlation between the current growth in the farming, forestry, and animal husbandry with growth with 4 lags, and between the current growth in the fisheries and the growth with 1 lag. This is resulted from the influence of past production decisions and expected returns on farmers’ production decisions, i.e., the expected outcomes based on past outputs will significantly influence farmers’ factor inputs in the current period. Another reason is that the biologically renewable resource stock of fisheries and forestry is not only influenced by biological factors, but also by human production activities, i.e., the availability of subsequent resources is directly affected by the amount of human collection in the current period. Thus, there is a significant negative relationship between the current and the lagged growth in the fishery and forestry sectors. Based on the experience of domestic and foreign scholars, the growth with 4 lags were incorporated into the dynamic panel model of farming, forestry, and animal husbandry, while the growth with 1 lag is added into the dynamic panel model of fishery sector.

**Table 2:** Correlation statistics.

Department	Variable	No lags	1 lag	2 lags	3 lags	4 lags	5 lags
Gross agricultural	*g*	1					
	L1*. g*	0.033*	1				
	L2. *g*	0.072***	0.026	1			
	L3. *g*	0.057***	0.064***	0.043**	1		
	L4. *g*	− 0.053***	0.056***	0.061***	0.036**	1	
	L5. *g*	− 0.022	− 0.059***	0.050***	0.051***	0.031*	1
Framing	*g*	1					
	L1*. g*	− 0.019	1				
	L2. *g*	− 0.022	− 0.023	1			
	L3. *g*	0.077***	− 0.031	− 0.016	1		
	L4. *g*	− 0.054***	0.077***	− 0.034*	− 0.019	1	
	L5. *g*	0.003	− 0.058***	0.075	− 0.039**	− 0.022	1
Forestry	*g*	1					
	L1*. g*	− 0.109***	1				
	L2. *g*	− 0.059***	− 0.114***	1			
	L3. *g*	− 0.049***	− 0.059***	− 0.110***	1		
	L4. *g*	− 0.300*	− 0.052***	− 0.066***	− 0.114***	1	
	L5. *g*	− 0.004	− 0.032*	− 0.055***	− 0.065***	− 0.112***	1
Animal husbandry	*g*	1					
	L1*. g*	0.032***	1				
	L2. *g*	0.023	0.014	1			
	L3. *g*	0.057***	0.006	0.035**	1		
	L4. *g*	− 0.012**	0.054***	− 0.005	0.086***	1	
	L5. *g*	− 0.042	− 0.025	− 0.042	− 0.025	0.085***	1
Fishery	*g*	1					
	L1*. g*	− 0.197***	1				
	L2. *g*	− 0.006	− 0.202***	1			
	L3. *g*	− 0.001	− 0.007	− 0.205***	1		
	L4. *g*	0.014	− 0.003	− 0.005	− 0.207***	1	
	L5. *g*	− 0.041**	0.015	− 0.009	− 0.007	− 0.209***	1

***, **, * indicate significant at the statistical level of 1%, 5%, and 10%, respectively.

## Results of the empirical analysis of the impact of temperature fluctuations on agricultural growth

### Impact of annual average temperature fluctuations on agricultural growth

This paper first analyzes the impact of temperature fluctuations on agricultural growth through Eq. (8). Based on the baseline regression model, temperature fluctuations with 10 lags are introduced to identify the impact of climate change more accurately. Tables 3 and 4 present estimated results of the impact of temperature fluctuations on growth in agriculture and its sectors, respectively, as well as the regression results with 1, 5 and 10 lags. Specifically, an increase in annual average temperature will significantly reduce the growth rate of gross agricultural output, meaning that a 1 °C increase in annual average temperature predicts a fall in the growth of gross agricultural output by 0.026 log points. This conclusion is consistent with the intuitive inference and existing studies, which reaffirms the representativeness and credibility of the data in this study. The increase in temperature will not only accelerate the evaporation of surface water and the occurrence of seasonal droughts, but also shorten the fertility stage of crops, leading to different degrees of decline in crop yield and plant biomass on the surface. On top of that, the increase in average temperature will further expand the scope of areas where pests were a terrible torment, and greatly increase the probability of the outbreak and spread of pests and diseases^35^. In terms of the growth in agricultural output, after the temperature fluctuations lags are introduced, it is clearly seen that the sum of the estimated coefficients of all lags is not significantly zero, despite interleaving positive and negative numbers. This indicates that temperature fluctuations have a significant negative cumulative effect on the growth of agricultural output in China.

**Table 3:** Estimated impact of annual average temperature changes on agricultural economic growth.

Variables	No lags		1 lag		5 lags		10 lags	
	Coef.	Std. Err.	Coef.	Std. Err.	Coef.	Std. Err.	Coef.	Std. Err.
Temp	− 0.026***	0.001	− 0.024***	0.001	− 0.022***	0.001	− 0.018***	0.001
L1: Temp			0.003***	0.000	0.003***	0.0000	− 0.005***	0.001
L2: Temp					− 0.011***	0.001	− 0.006***	0.001
L3: Temp					− 0.004***	0.001	0.002***	0.001
L1: Growth	0.061***	0.002	0.063***	0.002	0.062***	0.002	− 0.018***	0.0023
L2: Growth	0.069***	0.002	0.071***	0.001	0.080***	0.002	0.084***	0.0033
L3: Growth	0.009***	0.001	0.010***	0.001	0.017***	0.002	0.082***	0.002
L4: Growth	− 0.069***	0.001	− 0.068***	0.002	− 0.064***	0.002	− 0.021***	0.002
AR (1)	− 5.067***		− 5.068***		− 5.079***		− 5.248***	
AR (2)	− 1.077		− 1.033		− 0.920		0.913	
Sargan test	129.806		130.251		128.305		125.974	
Obs	3275		3275		3144		2489	

***, **, * indicate significant at the statistical level of 1%, 5%, and 10%, respectively.

**Table 4:** Estimated impact of average temperature on economic growth across agricultural sectors.

Variables	No lags	1 lag	5 lags	10 lags	No lags	1 lag	5 lags	10 lags
	Farming				Forestry			
Temp	− 0.027*** (0.001)	− 0.025*** (0.001)	− 0.025***(0.001)	− 0.020*** (0.001)	0.009*** (0.001)	0.014*** (0.001)	0.016*** (0.001)	0.018*** (0.002)
L1: Temp		− 0.009*** (0.001)	− 0.005*** (0.001)	− 0.013*** (0.001)		0.010*** (0.001)	0.010*** (0.001)	0.001 (0.002)
L2: Temp			− 0.022*** (0.001)	− 0.005*** (0.001)			− 0.009*** (0.001)	− 0.011*** (0.001)
L3: Temp			0.000 (0.001)	0.014*** (0.001)			− 0.009*** (0.001)	− 0.012*** (0.001)
Growth lags	Controlled	Controlled	Controlled	Controlled	Controlled	Controlled	Controlled	Controlled
Obs	3275	3275	3144	2489	3275	3275	3144	2489
	Animal Husbandry				Fisheries			
Temp	− 0.035*** (0.001)	− 0.033*** (0.001)	− 0.027*** (0.001)	− 0.022*** (0.001)	− 0.008*** (0.000)	− 0.010*** (0.000)	− 0.004*** (0.001)	− 0.014*** (0.001)
L1: Temp		0.002* (0.001)	0.002 (0.001)	− 0.008*** (0.001)		− 0.042*** (0.000)	− 0.014*** (0.001)	− 0.023*** (0.001)
L2: Temp.p			− 0.003*** (0.001)	− 0.012*** (0.001)			− 0.017*** (0.001)	− 0.009*** (0.001)
L3: Temp			− 0.009*** (0.001)	− 0.010*** (0.001)			− 0.024*** (0.001)	− 0.010*** (0.001)
Growth lags	Controlled	Controlled	Controlled	Controlled	Controlled	Controlled	Controlled	Controlled
Obs	3275	3275	3144	2489	3799	3668	3144	2489

***, **, * indicate significant at the statistical level of 1%, 5%, and 10%, respectively.

For each of the agricultural sector, whether it is farming, animal husbandry, or fisheries, the annual average temperature fluctuations will significantly contain their output growth. However, it is worth noticing that the effect of temperature fluctuations on output growth in the forestry sector is not consistent with the theoretical expectation. In contrast, increase in temperature has significantly increased the output growth. After the temperature fluctuations lags are introduced, it is found that the negative cumulative effect exists in all agricultural sectors such as farming and forestry, which means that the effect of temperature fluctuations on agricultural growth is significant in the long run, i.e., an increase in temperature will not only reduce the average output of agriculture but will also have a significant negative impact on agricultural productivity^36^. Differently, the average effect on output will disappear when the temperature returns to its prior state, while the effects on agricultural growth have always been apparent for long term. This confirms the theoretical analysis of this paper that an area may be caught in stagnation forever after experiencing temperature fluctuations during a time.

### Screening of variation in agricultural growth by temperature fluctuations

The impact of temperature fluctuations on agriculture is not homogeneous, and the identification of the sources of heterogeneity in the impact of temperature fluctuations can provide new ideas for following relative poverty management and regional synergistic development^37^. Considering this, this paper further adds interaction terms of temperature fluctuations with vulnerability and variability, and controls for fixed effects of time and region in turn, to further screen the heterogeneous effects and causes of temperature fluctuations on agricultural growth. Table 5 reports the results of model estimation based on the extension of Eq. (9). It is found that the negative impact of temperature increase on output growth in most agricultural sectors is more pronounced after the introduction of the interaction terms of vulnerability and variability. In terms of the heterogeneity of the impact of temperature fluctuations, although the direction of the estimated coefficients of the interaction term between vulnerability and temperature fluctuations is consistent with theoretical expectations, it shows significant differences from sector to sector. Overall, the increase in temperature will have a more severe negative impact on the gross agricultural output in relatively vulnerable areas, while the impact will be relatively smaller in areas with strong temperature fluctuations. In terms of agricultural sectors, the negative impact of temperature increase on economic output growth in relatively vulnerable areas is more pronounced in farming and forestry, yet less so in animal husbandry and fishery. However, the estimated results of the interaction term of temperature fluctuations are significantly positive for regions with different degrees of temperature fluctuations, while there is no significant gap between different agricultural sectors. This means that the impact of temperature increase on the level of agricultural growth in areas with severe temperature fluctuations is relatively small compared to areas with moderate temperature fluctuations, i.e., the marginal damage caused by temperature increase tends to diminish.

**Table 5:** Screening of variation in agricultural growth by temperature fluctuations.

Variables	Gross agricultural		Farming		Forestry		Animal Husbandry		Fisheries	
	Coef.	Std. Err.	Coef.	Std. Err.	Coef.	Std. Err.	Coef.	Std. Err.	Coef.	Std. Err.
Temp	− 0.027***	0.001	− 0.024***	0.002	0.002	0.002	− 0.046***	0.002	− 0.018***	0.001
Temp. $\times$ poor	− 0.005***	0.001	− 0.012***	0.002	− 0.032***	0.001	0.002***	0.002	0.002***	0.001
Temp. $\times$ worse	0.005***	0.001	0.004***	0.001	0.033***	0.001	0.016***	0.002	0.014***	0.001
L1: Growth	0.062***	0.001	− 0.045***	0.002	− 0.167***	0.001	− 0.039***	0.003	− 0.206***	0.000
L2: Growth	0.068***	0.001	0.038***	0.001	− 0.088***	0.001	0.003	0.002	–	–
L3: Growth	0.009***	0.001	0.045***	0.001	− 0.086***	0.001	0.029***	0.002	–	–
L4: Growth	− 0.069***	0.002	− 0.055***	0.001	− 0.062***	0.001	− 0.045***	0.002	–	–
AR (1)	− 4.770***		− 4.592***		− 2.871***		− 3.270***		− 2.684***	
AR (2)	0.438		− 0.576		0.098		− 0.431		0.304	
Sargan test	128.390		129.651		128.055		128.975		125.717	
Obs	3275		3275		3275		3275		3668	

***, **, * indicate significant at the statistical level of 1%, 5%, and 10%, respectively.

The above conclusions seem to be contrary to intuitive perceptions and theoretical hypotheses. However, what should not be ignored is that along with the structural adjustment of Chinese farmers’ sources of income, the share of income from agricultural operations in farmers’ total income has been shrinking. Relevant statistics show that the per capita wage income in rural areas of China was 5996.1 RMB in 2018, accounting for more than 40% of the per capita disposable income of farmers in that year, while the per capita wage income in developed eastern regions such as Shanghai and Zhejiang accounted for more than 60%, and the share of income from operations such as from agricultural operations was less than 30%.(Limitation of space forbids the exhibition of all lag results, and the table reports results of only the first three lags for each model.) The higher wage income means that farmers are relatively less dependent on agricultural production and has become less sensitive to temperature fluctuations and less willing to increase various production-oriented investments to counteract climate change. More importantly, farmers’ adaptive behavior also depends on the level technology supply from outside. Due to the lack of effective climate change coping technologies and measures, the demand for relevant coping technologies in the animal husbandry and fishery sectors has been so strong that it has topped the demand for climate change adaptation technologies for many years^38^.

At the same time, China has been facing severe pressure of temperature increase in recent years, and the average surface temperature rise is significantly higher than the global average for the same period^39^. Under the backdrop, although temperature increases still have a significant negative impact on agricultural growth, the marginal damage has been fading. Thus, the negative impact of temperature increase on the growth of agricultural output in areas with relatively moderate climate is more pronounced than in areas with drastic temperature fluctuations. From the estimated results of the amount of agricultural growth with lags, the estimated results show significant differences across sectors. Although the estimated coefficients of different lags show interleaving positive and negative numbers specifically, the lag of growth in gross agricultural output, farming, and animal husbandry show a positive leading effect on growth in the current period. As economically rational people, farmers will adjust their production decisions in the current period based on past business returns, and higher growth means that farmers can obtain relatively significant business returns, resulting in them continuously adjusting their production resources to maximize their returns. Although the same logic of farmers’ behavior applies to the forestry and fisheries sectors, unlike the farming sector, the access to resources in forestry and fisheries not only depends on the current factor inputs but is also constrained by the size of the existing resource stock. In most cases, higher growth is a result of higher levels of resource extraction^40^. Therefore, once the rate of resource exploitation exceeds the resource’s own resilience, the subsequent production level will naturally be limited.

## Re-examination based on different time intervals

### Divergence of temperature fluctuations and agricultural growth during the farming period

Table 6 illustrates the estimated results and sources of heterogeneity in the effects of average temperature fluctuations during the farming period on the growth of economic output in agriculture. To be more specific, the effects of average temperature fluctuations during the farming period are basically consistent with the results of the effects of an increase in the annual average. The increase in temperature will significantly reduce the growth rate of economic output, both in terms of gross agricultural output and economic output of farming, forestry, animal husbandry and fisheries respectively. Higher temperatures during the farming period will lead to water shortage and change the cycle of crop growth during the crop reproductive stage. As a result, a one-time 1ºC temperature increase during the farming period reduces growth of gross agricultural output by 0.025 log points. It is worth noticing that, unlike the estimated results for the average annual temperature, the increase in temperature during the farming period shows a more pronounced negative impact on the growth of economic output in fishery sector. Higher temperatures during the farming period, which is the main reproductive stage for various kinds of flora and fauna, will not only limit the formation of primary productivity of phytoplankton in the water body, negatively affecting fishery production, but also significantly reduce the oxygen level of the water body, thus increasing the probability of related diseases, and exerting negative impacts on the fishery output^41^.

**Table 6:** Estimated effects of changes in average temperature during the farming period on agricultural growth.

Variable	Gross agricultural		Farming		Forestry		Animal Husbandry		Fishery	
	(1)	(2)	(1)	(2)	(1)	(2)	(1)	(2)	(1)	(2)
Temp	− 0.025*** (0.000)	− 0.026*** (0.001)	− 0.027** (0.001)	− 0.030*** (0.001)	− 0.023*** (0.001)	− 0.029*** (0.003)	− 0.025*** (0.001)	− 0.025*** (0.002)	− 0.032*** (0.000)	− 0.033*** (0.001)
Temp. $\times$ poor		− 0.011*** (0.001)		− 0.007*** (0.001)		0.004 (0.003)		− 0.014*** (0.001)		− 0.006*** (0.001)
Temp. $\times$ worse		0.011*** (0.002)		0.013*** (0.001)		0.009*** (0.002)		0.002*** (0.000)		0.003*** (0.001)
L1: Growth	0.067*** (0.002)	0.065*** (0.001)	0.041*** (0.001)	0.044*** (0.002)	− 0.171*** (0.056)	− 0.172*** (0.001)	− 0.025*** (0.003)	− 0.029*** (0.002)	− 0.208*** (0.000)	− 0.207*** (0.000)
L2: Growth	0.073*** (0.001)	0.072*** (0.002)	0.043*** (0.002)	0.041*** (0.001)	− 0.090** (0.040)	− 0.092*** (0.001)	0.008*** (0.002)	0.005*** (0.001)		
L3: Growth	0.005*** (0.001)	0.005*** (0.001)	0.046*** (0.001)	0.045*** (0.001)	− 0.088*** (0.050)	− 0.089*** (0.001)	0.025*** (0.001)	0.024*** (0.001)		
L4: Growth	− 0.072* (0.001)	− 0.071*** (0.002)	− 0.056*** (0.001)	− 0.057*** (0.001)	− 0.064*** (0.040)	− 0.064*** (0.001)	− 0.042*** (0.001)	− 0.040*** (0.001)		
Cons.	0.069*** (0.001)	0.054*** (0.001)	0.068*** (0.001)	0.057*** (0.002)	0.114*** (0.012)	0.096*** (0.002)	0.070*** (0.001)	0.049*** (0.002)	0.117*** (0.002)	0.116*** (0.001)
AR (1)	− 4.998***	− 4.983*** 【】	− 4.551***	− 4.529***	− 2.737	− 2.883***	− 3.235***	− 3.214***	− 3.085***	− 3.088***
AR (2)	− 1.124	− 1.136	− 0.03	− 0.634	0.065	0.085	− 0.457	− 0.459	− 1.683	− 1.689
Sargan test	128.864	127.357	− 0.608	130.286	127.392	125.702	128.573	128.696	129.518	128.513
Obs	3275	3275	3275	3275	3275	3275	3275	3275	3668	3668

(1) The values in parentheses are robust standard errors.

(2) ***, **, * indicate significant at the statistical level of 1%, 5%, and 10%, respectively.

In terms of the heterogeneous effects on output growth in agriculture, temperature fluctuations during the farming period show a consistent impact on output growth in other sectors except for forestry. To be more specific, the output growth of farming, animal husbandry and fishery in relatively vulnerable areas are more vulnerable to the rising temperature during the farming period. In contrast, the increase in temperature during the farming period has not severely affected the output growth of forestry in relatively vulnerable areas. Compared with perennial forestry vegetation, most crops are more sensitive to temperature fluctuations during the farming period when warm climate favors many types of plants. On top of that, the coefficient of the interaction term with the degree of temperature changes is still significantly positive, indicating that the marginal damage of temperature fluctuations on the growth of agricultural output tends to diminish. In the areas with stronger temperature fluctuations, the effects of higher temperature are relatively less during the farming period.

### Divergence of temperature fluctuations and agricultural growth during the fallow period

Table 7 presents the estimated results of the impact of average temperature fluctuations during the fallow period on output growth in each agricultural sector, which is slightly different from the estimates of temperature changes during the annual and the farming period. Although increase in average temperature during the fallow period will significantly constrain the level of growth in the farming and animal husbandry sectors, this conclusion does not apply to the forestry and fishery sectors. In the case of the farming, lower temperatures during the fallow period are effective in reducing the number of pests and diseases in the soil, while a warm winter will significantly increase the probability of pest outbreak in the following year, which will have a negative impact on agricultural production^42,43^. In comparison, in the forestry and fishery sectors, the increase in temperature during the fallow period creates more suitable natural conditions for plants and animals to survive the winter, which effectively reduces the cold-related mobility of poultry and fish seedlings while extending the length of the reproductive stage of plants.

**Table 7:** Estimated impact of average temperature fluctuations on agricultural growth during the fallow period.

Variable	Gross agricultural		Farming		Forestry		Animal Husbandry		Fishery	
	(1)	(2)	(1)	(2)	(1)	(2)	(1)	(2)	(1)	(2)
Temp	− 0.004*** (0.000)	− 0.002*** − 0.001	− 0.004*** (0.000)	− 0.006*** (0.001)	0.017*** (0.000)	0.013*** (0.001)	− 0.009*** (0.000)	− 0.009*** (0.001)	0.002*** (0.000)	0.010*** (0.000)
Temp. $\times$ poor		0.009*** (0.001)		0.014*** (0.001)		− 0.007*** (0.001)		0.012*** (0.001)		− 0.015*** (0.001)
Temp. $\times$ worse		− 0.012*** (0.001)		− 0.010*** (0.001)		− 0.009*** (0.001)		− 0.010*** (0.001)		− 0.002*** (0.001)
L1: Growth	0.074*** (0.001)	0.076*** (0.001)	0.033*** (0.001)	0.032*** (0.002)	− 0.167*** (0.001)	− 0.166*** (0.001)	0.027*** (0.002)	0.025*** (0.002)	− 0.204*** (0.000)	− 0.204*** (0.000)
L2: Growth	0.076*** (0.001)	0.076*** (0.001)	0.047*** (0.001)	0.050*** (0.002)	0.087*** (0.001)	− 0.086*** (0.001)	0.011*** (0.001)	0.011*** (0.002)		
L3: Growth	0.012*** (0.001)	0.012*** (0.001)	0.053*** (0.001)	0.054*** (0.001)	0.084*** (0.001)	− 0.085*** (0.001)	0.034*** (0.001)	0.035*** (0.001)		
L4: Growth	− 0.066*** (0.001)	− 0.067*** (0.001)	− 0.052*** (0.001)	− 0.051*** (0.001)	− 0.061*** (0.001)	− 0.059*** (0.001)	− 0.040*** (0.001)	− 0.040*** (0.002)		
Cons.	0.061*** (0.001)	0.072*** (0.001)	0.062*** (0.001)	0.072*** (0.001)	0.075*** (0.001)	0.071*** (0.002)	0.070*** (0.001)	0.079*** (0.001)	0.102*** (0.001)	0.098*** (0.001)
AR (1)	− 5.028***	− 5.085***	− 4.606***	− 4.620***	− 2.887***	− 2.892***	− 3.275***	− 3.281***	− 3.087***	− 3.091***
AR (2)	− 0.962	− 1.013	− 0.474	− 0.500	0.114	0.096	− 0.412	− 0.454	− 1.610	− 1.629
Sargan test	128.668	128.993	127.876	126.623	127.980	122.355	129.248	127.932	129.930	129.269
Obs	3275	3275	3275	3275	3275	3275	3275	3275	3668	3668

(1) The values in parentheses are robust standard errors.

(2) ***, **, * indicate significant at the statistical level of 1%, 5%, and 10%, respectively.

In terms of the fractionation of agricultural growth, the coefficients of the interaction term between temperature fluctuations and vulnerability shows the opposite results. In terms of the output growth of farming and animal husbandry, the relatively vulnerable areas are less affected by temperature increases during the fallow period. This may be related to structural differences in rural household income, or due to the lack of off-farm employment opportunities for low-income farmers, with the end of farming production activities indicating a period of relative abundance of agricultural labor. During this period, considering the relatively low labor cost, farmers will be induced to conduct labor-biased adaptation behaviors, such as collecting dry branches, fallen leaves, and deep tilling the land, to address the impacts of climate change^44–46^. It is worth noticing that although the increase in temperature during the fallow period is beneficial to the growth of economic output in the forestry and fishery sectors, the relatively vulnerable areas do not benefit equally. On the one hand, with a lower income, farmers have significant weaknesses in a string of related factors (e.g., production investment, technology, human capital), which in turn lead to a growing gap in agricultural productivity between the rich and poor areas; on the other hand, sound agricultural infrastructure has an obvious multiplier effect on commercialization of agricultural products. Therefore, the failing infrastructure in vulnerable areas will inevitably limit the commercialization of their agricultural products, miring the products in a passive situation where the strong will on price upturn cannot be translated into sales. In addition, the heterogeneous impact of temperature fluctuations during the fallow period on areas with different degrees of variability is also worthy of attention. Different from the above results of temperature fluctuations, the negative impact of temperature increase is more pronounced in areas with strong temperature fluctuations, both in terms of gross agricultural output and output of each agricultural sector. Compared with the higher temperatures baseline during the farming period, the fallow period in all regions of China means the arrival of winter, when plants and animals with a lower temperature baseline tend to be more sensitive to the temperature increase. Therefore, the marginal damage to agricultural growth will rise with the increase in temperature.

## Basic conclusions and policy implications

Climate change, characterized by the rising temperature, is one of the inevitable severe challenges for the agricultural development of China. The effect that the negative impact of temperature rising, and the implementation of related policies have on the narrowing development gap among regions as well as alleviating relative poverty is non-negligible. Based on the agricultural production data of 131 major cities in China from 1991 to 2018, this paper conducts an empirical study on the differences in the impact of the rising temperature on the agricultural economic growth in different regions from two-time dimensions, the farming period and fallow period. Based on this, the marginal contributions of this paper are firstly, based on the current development situation of relative poverty governance, the research object is changed from the "stock" of output to the "increment" of output, and the impact of climate change on the economic growth of agriculture as a whole and each sector is assessed. Secondly, the historical average temperature of each region is used as a benchmark to reconsider the temperature change in each region. More importantly, this paper further explores the sources of heterogeneity in the impact of climate change on agricultural economic growth from the perspectives of marginal impact and adaptive capacity and conducts an empirical analysis with the help of a large sample of data from 131 cities across China over a long-time span to improve the accuracy and generalizability of the research findings. The study is intended to improve the precision and generalizability of the findings. The research findings are as follows:

First, the rising annual average temperature will reduce the output growth level of various agricultural sectors, and the negative impact of the rising temperature is more obvious in those relatively vulnerable areas. More importantly, the restrictions on the agricultural economic growth that the rising temperature brings mainly come from its influence on the efficiency of production. However, this negative impact is just irreversible, which will further widen the gap of the agricultural economic output among regions; second, the impact of the rising temperature on agricultural economic growth is mainly concentrated in the farming period, and its marginal damage to agricultural economic growth shows a downward trend. Third, the rising temperature in the agricultural fallow period also has a non-negligible impact on agricultural economic growth in likewise, and there are obvious differences among different agriculture sectors. Although the rising temperature in the fallow period will significantly reduce the growth level of economic output for the agriculture and animal husbandry sector, the strike that those relatively vulnerable areas suffer is relatively small. In the meantime, the increasing temperature during the slack period will contribute to improving the growth level of economic output of forestry and fishery departments, while those relatively vulnerable areas haven’t obtained equalized positive benefits from the rising temperature. It is worth noting that the impact of the rising temperature on agricultural economy during the agricultural fallow period is still in elementary stage, that is, the degree of marginal damage shows a growing trend with the intensification of temperature fluctuation.

The focus of relevant departments has always been how to reduce the impact of temperature fluctuations on agriculture^47^. The policy implication of this paper lies in providing a more targeted reference for subsequent governance of temperature fluctuations, as well as considering the historical mission of narrowing development gap among regions and alleviating relative poverty.

On the one hand, in terms of slowing down the temperature rise, we should continue to strengthen the implementation of the conference spirit of energy saving and emission reduction proposed by the World Climate Conference and promote the green and high-quality development of agricultural economy. At the same time, we should accelerate the construction of the prediction and early warning system of the high temperature meteorological department, vigorously develop intelligent agricultural meteorological services, and provide timely and accurate weather warning information for the government and farmers. Although the marginal damage of rising temperatures during the farming period is on a downward trend, the impact of rising temperatures on agriculture during the fallow period is gradually increasing. Therefore, it is essential to import or select and breed more resistant crop varieties to cope with the variability of climate change conditions corresponding to different regions and to better secure agricultural output.

On the other hand, in terms of enhancing the adaptive capacity to cope with rising temperatures, the supply level of adaptive technologies for rising temperatures, especially for the fallow period, should be further stimulated, based on measures such as selected seeds, increased application of organic fertilizers and controllable seedlings, and through agricultural control, physical control and biological control, etc., the time range covered by adaptive technologies should be expanded to realize the change from seasonal technologies for agricultural production to annual technologies for production. Based on this, it may be a comprehensive reduction of the impact of temperature changes on agricultural output. At the same time, we should consider the differences between agricultural sectors and choose a differentiated technology supply system and focus. More importantly, we should focus on the universality of technological progress. By constructing a reasonable technology subsidy policy, gradually strengthening the construction of agricultural infrastructure and guiding the transfer of advanced technologies to vulnerable areas through knowledge dissemination and technology promotion, we can effectively improve the coping ability of farmers in vulnerable areas and encourage them to adopt adaptive behaviors to reduce the negative impacts caused by temperature rise, gradually build a perfect agricultural weather disaster defense system, and avoid the gap between regional agricultural economic development^48^.

We summarize the limitations of this study and the entry points for future research by combining the results of existing research as follows. In terms of temperature variation changes, this research applied is the annual average temperature, i.e., the average temperature of each month of the year in the region is summed up and divided by 12 to represent an arithmetic mean. And there is no doubt that this will bring more error and unobservability to the data processing results. Therefore, the authors will further contact the China Meteorological Administration and others in the next section to use cumulative temperature as the main core explanatory variable, which is the sum of daily average temperature during a year that is greater than or equal to a certain critical temperature duration, and is an essential indicator to study the relationship between temperature and agricultural crop production and development, which is of significance for agricultural economic development and services.

In the scope of regional data application, the article applies urban data, but it’s possible that, as the reviewer stated, agricultural production is a more refined industry and there is a large regional heterogeneity in different cities, which may lead to different temperature variations in one city. When the range of regional temperature fluctuations is large, it will have certain negative effects on the development of agricultural economy, and thus a major bias will occur. Therefore, in the next study, the team will go further into the specific counties or towns in the city and cooperate with the grassroots government to obtain more micro and specific data, so that the research results can be more in line with the actual agricultural economic development.

## References

[1] Poppenborg P, Koellner T (2013). Do attitudes toward ecosystem services determine agricultural land use practices? An analysis of farmers’ decision-making in a South Korean watershed. Land Use Policy.

[2] Chavas DR, Thomson IRC (2009). Long-term climate change impacts on agricultural productivity in eastern China. Agric. For. Meteorol..

[3] Lü Y, Fu B, Feng X (2012). A policy-driven large scale ecological restoration: quantifying ecosystem services changes in the Loess Plateau of China[J]. PLoS ONE.

[4] Qu C, Shao J, Shi Z (2020). Does financial agglomeration promote the increase of energy efficiency in China?. Energy Policy.

[5] Zhang S, Wang Y, Hao Y (2021). Shooting two hawks with one arrow: Could China's emission trading scheme promote green development efficiency and regional carbon equality?. Energy Economics.

[6] Piao S, Ciais P, Huang Y (2010). The impacts of climate change on water resources and agriculture in China. Nature.

[7] evidence from China (2016). Chen, S., X. Chen*, and J. Xu, Impacts of climate change on agriculture. J. Environ. Econ. Manag..

[8] Trinh TQ, Rañola RF, Camacho LD (2018). Determinants of farmers’ adaptation to climate change in agricultural production in the central region of Vietnam[J]. Land Use Policy.

[9] Su Y, Gabrielle B, Makowski D (2021). The impact of climate change on the productivity of conservation agriculture[J]. Nat. Clim. Chang..

[10] Kurashima N, Fortini L, Ticktin T (2019). The potential of indigenous agricultural food production under climate change in Hawaiʻi[J]. Nature Sustain..

[11] Mase AS, Gramig BM, Prokopy LS (2017). Climate change beliefs, risk perceptions, and adaptation behavior among Midwestern US crop farmers [J]. Clim. Risk Manag..

[12] Tambo JA, Abdoulaye T (2012). Climate change and agricultural technology adoption: The case of drought tolerant maize in rural Nigeria[J]. Mitig. Adapt. Strat. Glob. Change.

[13] Chen S, Gong BL (2020). Response, and adaptation of agriculture to climate change: evidence from China. J. Dev. Econ..

[14] Schoengold K, Ding Y, Headlee R (2015). The impact of AD HOC disaster and crop insurance programs on the use of risk-reducing conservation tillage practices. Am. J. Agr. Econ..

[15] Solomon H, Paulina O (2019). The distribution of environmental damages [J]. Rev. Environ. Econ. Policy.

[16] Barbier, E.B., & Hochard, J.P. The impacts of climate change on the poor in disadvantaged regions[J]. Rev. Environ. Econ. Policy (2020).

[17] Diffenbaugh NS, Burke M (2019). Global warming has increased global economic inequality[J]. Proc. Natl. Acad. Sci..

[18] Lake IR, Hooper L, Abdelhamid A (2012). Climate change and food security: health impacts in developed countries[J]. Environ. Health Perspect..

[19] Kumar S, Khanna M (2019). Temperature, and production efficiency growth: empirical evidence. Clim. Change.

[20] Li Y, Xiong W, Hu W (2015). Integrated assessment of China’s agricultural vulnerability to climate change: a multi-indicator approach[J]. Clim. Change.

[21] Cui Q, Ali T, Xie W (2022). The uncertainty of climate change impacts on China’s agricultural economy based on an integrated assessment approach[J]. Mitig. Adapt. Strat. Glob. Change.

[22] Hsiang SM (2010). Temperatures and cyclones strongly associated with economic production in the Caribbean and Central America. Proc. Natl. Acad. Sci..

[23] Melissa D, Benjamin F, Benjamin AO (2014). What do we learn from the weather? The new climate economy literature. J. Econ. Liter..

[24] Melissa D, Benjamin F, Benjamin AO (2012). Temperature shocks and economic growth: evidence from the last half century. Am. Econ. J. Macroecon..

[25] Carleton TA, Hsiang SM (2016). Social and economic impacts of climate. Science.

[26] Ostaev GY, Kondratyev DV, Kravchenko NA (2020). Crisis identification and development of crisis management algorithm in the agricultural sector[J]. Amaz. Invest..

[27] Yu B, Liu F, You L (2012). Dynamic agricultural supply response under economic transformation: A case study of Henan. China. Am. J. Agric. Econ..

[28] Ebi, K.L., Hess, J.J., & Watkiss, P. Health risks and costs of climate variability and change [J]. *Disease Control Priorities* **7** (2017). 30212118

[29] Bond S, Leblebicioǧlu A, Schiantarelli F (2010). Capital accumulation and growth: A new look at the empirical evidence. J. Appl. Economet..

[30] Dell M, Jones BF, Olken BA (2012). Temperature shocks and economic growth: Evidence from the last half century. Am. Econ. J. Macroecon..

[31] Howard J, Sutton GA, Herr D (2017). Clarifying the role of coastal and marine systems in climate mitigation. Front. Ecol. Environ..

[32] Schlenker W, Roberts MJ (2009). Nonlinear temperature effects indicate severe damages to US crop yields under climate change. Proc. Natl. Acad. Sci..

[33] Janjua AA, Aslam M, Sultana N, Batool Z (2021). Identification of climate induced optimal rice yield and vulnerable districts rankings of the Punjab. Pakistan. Sci. Rep..

[34] Letta M, Richard SJT (2019). Weather, climate and total factor productivity. Environ. Resour. Econ..

[35] Skendžić S, Zovko M, Živković IP (2021). The impact of climate change on agricultural insect pests[J]. Insects.

[36] Ortiz-Bobea A, Ault TR, Carrillo CM (2021). Anthropogenic climate change has slowed global agricultural productivity growth [J]. Nat. Clim. Chang..

[37] Clay N, King B (2019). Smallholders’ uneven capacities to adapt to climate change amid Africa’s ‘green revolution’: Case study of Rwanda’s crop intensification program[J]. World Dev..

[38] Princiotta FT, Loughlin DH (2014). Global climate change: The quantifiable sustainability challenge[J]. J. Air Waste Manag. Assoc..

[39] Li B, Chen Y, Shi X (2012). Why does the temperature rise faster in the arid region of northwest China?. J. Geophys. Res. Atmos..

[40] Shang X, Guo QH (2010). Analysis of behaviors of part-time peasant household based on rational economic man hypothesis[J]. J. Jilin Agric. Univ..

[41] Castellani, C., & Edwards, M. eds. Marine Plankton: A practical guide to ecology, methodology, and taxonomy. Oxford University Press, (2017).

[42] Ciscar JC, Iglesias A, Feyen L (2011). Physical and economic consequences of climate change in Europe. Proc. Natl. Acad. Sci..

[43] Hsiang S, Kopp R, Jina A (2017). Estimating economic damage from climate change in the United States. Science.

[44] Erda L, Wei X, Hui J (2005). Climate change impacts on crop yield and quality with CO2 fertilization in China. Philos. Trans. R. Soc. B Biol. Sci..

[45] Aggarwal PK (2008). Global climate change and Indian agriculture: Impacts, adaptation, and mitigation. Indian J. Agric. Sci..

[46] Gerrit H (2015). The evolution of the evidence bases for observed impacts of climate change. Curr. Opin. Environ. Sustain..

[47] Liying W, Shuyu D, Houping LIU (2020). Organic connection between small farmers and modern agriculture development based on farmers’ cooperatives in China. Asian Agric. Res..

[48] Burke M, Hsiang SM, Miguel E (2015). Global Non-Linear effect of temperature on economic production. Nature.

